# Magnitude and Implications of Interfraction Variations in Organ Doses during High Dose Rate Brachytherapy of Cervix Cancer: A CT Based Planning Study

**DOI:** 10.1155/2014/687365

**Published:** 2014-02-03

**Authors:** Santam Chakraborty, Firuza D. Patel, Vijay M. Patil, Arun S. Oinam, Suresh C. Sharma

**Affiliations:** Department of Radiotherapy, Regional Cancer Centre, Postgraduate Institute of Medical Education and Research, Chandigarh 160012, India

## Abstract

*Background*. Quantifying the interfraction dose variations in the organs at risk (OAR) in HDR intracavitary brachytherapy (HDR ICBT). *Methods*. Rectum and bladder were contoured in 44 patients of cervical carcinoma on CT after each fraction of HDR ICBT (9 Gy/2 fractions). Interfraction dose variations (VAR_act_) were calculated. Rigid image registration of consecutive fraction images allowed quantification of the hypothetical variation in dose (VAR_hypo_) arising exclusively due to changes in applicator placement and geometry. VAR_hypo_ was regressed against the VAR_act_ to find out to what extent the applicator variation could explain the VAR_act_ in the OAR. The rest of the variation was assumed to be due to organ deformation. *Results*. The VAR_act_ in the dose to 2 cc of bladder and rectum were 1.46 and 1.16 Gy, respectively. Increased dose was seen in 16 and 23 patients in the subsequent fraction for bladder and rectum, respectively. Doses to OAR would have exceeded constraints in 16% patients if second fraction was not imaged. VAR_hypo_ explained 19% and 47% of the VAR_act_ observed for the bladder and rectum respectively. *Conclusions*. Significant interfraction variations in OAR doses can occur in HDR ICBT. Organ deformations are mostly responsible for this variation.

## 1. Background

Intracavitary brachytherapy (ICBT) forms an integral part of management of cervical carcinoma. While high dose rate ICBT (HDR ICBT) has become popular due to its logistical advantages over low dose rate ICBT (LDR ICBT), it also necessitates dose fractionation in order to reduce normal tissue complications [1]. This results in inadvertent changes in the position/geometry of the applicator. In addition there are interfraction deformations in organs at risk (OAR) due to movement, shape changes, and variable filling of these hollow organs. These in turn result in organ dose variations, which have important implications in dose reporting.

In the past several authors had described the interfraction variation in applicator geometry as well as that of the organ point doses using data from orthogonal radiographs [2–9]. Recent advances in technology have allowed the use of volumetric imaging in gynecological brachytherapy planning [10, 11]. As a result greater information is available on the volumetric doses received by these OAR themselves. Data from some of the recent series have highlighted the problem of interfraction dose variation in HDR brachytherapy using volumetric imaging modalities [12–14].

Interfraction variations in the doses to these OAR may arise as a consequence of changes in two factors, applicator position/geometry changes on one hand and organ deformation on the otherhand. Of these two, applicator positioning and geometry depend largely on the person performing the procedure and thus can be controlled to a certain extent by using standardized application techniques.

The study was designed in order to answer the following three specific questions.

1. What was the magnitude of interfraction dose variation to the OAR in our patient population?
2. How harmful was this variation in terms of excess “unrecognized” dose to the OAR if the subsequent fraction was not imaged?
3. How much of the variation could be accounted for by the changes in applicator position/geometry versus deformation of the OAR concerned?

## 2. Methods

A retrospective analysis was undertaken in 44 patients of cervical carcinoma, who underwent CT based planning for both fractions of ICBT between June 2007 and June 2008. All patients were treated with external beam radiation therapy to a dose of 46 Gy in 23 fractions over four and half weeks, followed by two fractions of HDR ICBT one week apart (9 Gy to point A in each fraction). Informed consent about the intracavitary procedure and the CT scanning procedure had been taken individually. Special ethical clearance was not required keeping in mind the retrospective nature of this analysis.

Metallic tandem ovoid type HDR microSelectron after-loading applicators designed according to the Manchester geometry (Nucletron B.V., The Netherlands) were used. All applications were performed under general anesthesia (GA) and involved placement of an intrauterine tandem and vaginal ovoids. Vaginal packing with dry gauze was done to fix the applicator in position and to displace the bladder and rectum away from the vaginal applicators. A stitch was placed on the vulva to secure the system in place.

As a departmental policy we use the same applicator in both the fractions. Three different tandem lengths were used, 6 cm (22 patients), 5 cm (21 patients), and 4 cm (1 patient). Full ovoids (diameter = 2 cm) were used in 31 patients while half ovoid pairs were used in 13 patients. The half ovoid (radius = 1 cm) applicator (13 patients) has the facility for setting flexible tandem lengths and for this reason the ovoid can be fixed at variable positions along with the desired tandem length. In the other applicators the craniocaudal movement of the ovoids is restricted by a fixed coupling as the tandem length is fixed.

CT simulation was done on helical CT (Lightspeed VXR 16, GE Medical Systems, Waukesha, USA) without any dummies inside the applicator. Prior to the scan, 7 cc of radio-opaque dye (1 : 4 dilution) was instilled in the balloon of Foley's catheter, which was then pulled snugly against the trigone of bladder. A specialized scan protocol utilizing differential slice thickness was used in order to ensure the best visualization of the applicator. The slice thickness was 2.5 mm from the tip of the tandem to the superior surface of the ovoids and 0.65 mm from the superior surface of the ovoids to the inferior surface. The scanned volume extended from 2 cm above the tip of the applicator to 5 cm below the inferior surface of the ovoids.

The Advantage CT/MR Fusion (version 1.2) software [15] fraction (CT_1_) was registered on the second fraction image (CT_2_) with the after-loading method. This involved finding an optimal rigid transformation using an initial automatic setup, based on the extraction of common contours, and a manual registration, based on bony landmarks. These bony landmarks were the pubic tubercle, right ischial tuberosity, and the left ischial spine [15]. The best transform between these 3 pairs of points was computed by the software using a gradient descent algorithm. Accuracy of the registration thus performed was measured using the registration score calculated by the software and visual check using the correlated cursor provided by the software. The registration score error represented the remaining distance in mm between the two points after registration. For the present study we used the registered image set when the maximum error was less than two mm. Bladder was contoured as a solid organ till the start of the urethra. Rectum was contoured as a solid organ from tip of the coccyx till the sigmoid flexure [10].

**Figure 1:**
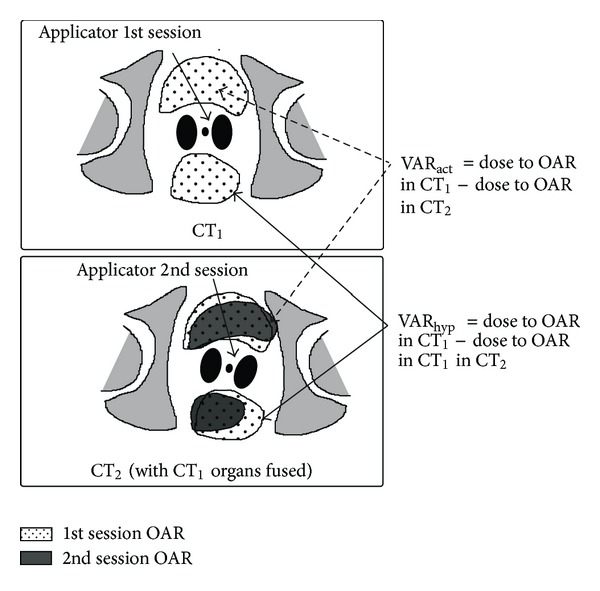
Sketch showing the concept behind the study. In the second fraction CT the registered organs of the 1st fraction maintain their positions with respect to the bony anatomy after image registration. The applicator has however changed its position and geometry in the second fraction image. The dose to the 1st fraction organs when calculated from the 2nd fraction CT is the hypothetical dose that will result if the only change between the two fractions was the change in applicator position and geometry. The actual organ position in the 2nd fraction is depicted in dark grey.

As a result of the rigid registration, the contours of the OAR in the CT_1_ could be transferred onto CT_2_ maintaining same coordinates with respect to the bony anatomy as in the actual CT_1_ (Figure 1). CT_1_ thus contained the contours of the OAR for the first fraction only, while CT_2_ had the contours of the OAR of both fractions. This registration allowed us to generate the hypothetical situation where we could evaluate the dose variation that would have resulted if applicator variation was solely responsible for the actual variation as described below.

Both the images and the fusion structure sets were transferred to the PLATO Sunrise brachytherapy planning workstation for planning and calculation (Nucletron B.V., The Netherlands). Applicator reconstruction was done in the PLATO sunrise workstation using the Multi-Planar Reconstruction (MPR) view. In order to delineate a particular channel, the axis of the image was manipulated so that it passed through the desired channel. This provided a display of the reconstructed CT images in the desired plane. In this view the tip and channel could be easily demarcated and reconstructed following the curvature of the catheters [10]. The dwell positions in the tandem and the ovoids included an offset of 4 mm which was calculated on the basis of autoradiographs acquired during the quality assurance process.

A dose of 9 Gy was prescribed to point A (defined as pert the ICRU 38 definition) for each fraction [12]. In our institute the same standard loading pattern is used for both fractions unless OAR dose constraints are exceeded. In such cases, we perform a graphical optimization keeping in mind that the point A doses remain constant as far as possible. Therefore in the present study we used standard loading in both fractions without additional optimization in order to quantify the dose variation between two fractions in our setting. The use of the same loading pattern for all cases also helped in eliminating the influence of variable optimization on dose variation across fractions.

Volumes of the delineated rectum and bladder were calculated in each individual fraction. The maximum point doses to these organs, along with the minimum dose in the most irradiated tissue adjacent to the applicator for 0.1, 1, and 2 cm^3^ (*D*_0.1 cc_, *D*_1 cc_, and *D*_2 cc_) volumes, were noted for both fractions. Due to artifacts arising from use of metallic applicators we could not delineate the organ walls reproducibly and hence doses to 5 and 10 cm^3^ are not being reported [14]. However as the *D*_2 cc_ doses are considered most relevant clinically we restricted further analysis to this volume bin [11]. Because of differing biological effectiveness, the *D*_2 cc_ in each brachytherapy fraction were converted to the equivalent dose at 2 Gy per fraction (EQD2) using the formula EQD2 = *D*(1 + *D*/(*α*/*β*))/1 + 2/(*α*/*β*), assuming an *α*/*β* ratio of 3. The actual cumulative EQD2 (in Gy_*α*/*β*=3_) for 2 cc volume was then calculated by adding the EQD2 of the two fractions with that of the external beam radiotherapy course (which was 46 Gy).

### 2.1. Study Design and Statistical Analysis

In order to answer the questions posed at the start we proceeded in three steps. First the actual dosimetric and volumetric variations observed for the OAR were calculated. Mean and 95% confidence intervals (CI) of the mean were calculated for each parameter. Paired *t*-test was used to compare the difference in means for these dose volume parameters between two fractions. All tests were two tailed and *P* values of <0.05 were taken as significant.

The utility of the second fraction imaging lies in its ability to pick up unintended overdose of the OAR that results if the first fraction dose plan is used in subsequent fractions. For this purpose the dose received by the OAR in the first fraction was doubled, mimicking the situation where the doses in both fractions were assumed to be the same. A “predicted” cumulative EQD2 was then calculated from this doubled dose. The difference between this predicted EQD2 and the cumulative EQD2 actually received by the OAR over the two fractions gave us the magnitude of the error introduced by assuming that the first fraction OAR doses remained the same in the subsequent fraction. The percent of patients, who would have exceeded the dose constraints for bladder (cumulative EQD2 > 90 Gy_*α*/*β*=3_) and rectum (cumulative EQD2 > 75 Gy_*α*/*β*=3_), was calculated [10]. Furthermore the percent difference in the predicted EQD2 and the actual EQD2 was then plotted on a population pyramid which showed at a glance the frequency and magnitude of this error.

In order to quantify the relative importance of the applicator geometry/position change versus organ deformation in producing the actual dose variation observed, we had to separate their individual contribution in the actual dose variation. For this purpose we assumed a hypothetical situation where no deformation in the OAR had occurred and the dose variation in the second fraction was solely due to the changes in applicator geometry and positioning. This was made possible by the use of the rigid image fusion technique where the bony anatomy was taken as the frame of reference as shown in Figure 1.

For each patient three dose parameters were determined as described in the following:

1. dose to the OAR of CT_1_ in CT_1_,
2. dose to the OAR of CT_2_ in CT_2_,
3. dose to the OAR of CT_1_ in contours registered in CT_2_. In CT_2_ doses to two sets of contours of each organ (fused and actual) were noted.

The difference between the dose to OAR between that noted on CT_1_ and CT_2_ was the actual dose variations observed (VAR_act_). The difference between the dose to the OAR in the CT_1_ and the dose noted for the fused contour of that organ in CT_2_ gave us the hypothetical variation (VAR_hyp_), which would have resulted if only the applicator placement varied between the two fractions (Figure 1). The following equations illustrate the formula used for bladder *D*_2 cc_ to calculate each of the above:

(1) VARact Bladder=Dose to Bladder of CT1 in CT1−Dose to Bladder of CT2 in CT2,VARhypo Bladder=Dose to Bladder of CT1 in CT1−Dose to Bladder of CT1 in CT2.

VAR_hypo_ for *D*_2 cc_ of rectum and bladder was then regressed on the VAR_act_ for the *D*_2 cc_ for the respective organs between the two fractions. The coefficient of determination obtained from this regression equation was then used for calculating the proportion of the actual dose variations (VAR_act_) that could be explained by the variation in the applicator position only (VAR_hypo_). Any remaining difference could have arisen only due to organ deformation and thus we were able to quantify which of the two factors was more important in producing the actual dose variation observed.

## 3. Results

The mean contoured rectal volumes for the 1st and 2nd fractions were 43.24 and 44.51 cm^3^, respectively, while the respective volumes for bladder were 84.14 and 78.17 cm^3^. The mean difference in the rectal volume in two fractions was 9.16 cm^3^ (standard deviation: 7.76 cm^3^; 95% CI of mean: 6.80–11.50 cm^3^), while that for the bladder was 30.63 cm^3^ (standard deviation: 38.14 cm^3^; 95% CI of mean: 19.03–42.23 cm^3^). These differences were statistically insignificant (*P* value: 0.49 and 0.42 for rectum and bladder, resp.).

The maximum variation observed in the bladder *D*_2 cc_ was 6.16 Gy in one patient. The mean EQD2 bladder *D*_2 cc_ was 25.27 Gy_*α*/*β*=3_ (95% CI: 22.40–28.13) and 23.82 Gy_*α*/*β*=3_ (95% CI: 21.06–26.56) in the first and second fraction ICBT respectively. The maximum variation in the rectal *D*_2 cc_ was 3.86 Gy in one patient. The mean EQD2 for the rectal *D*_2 cc_ was 9.92 Gy_*α*/*β*=3_ (95% CI: 8.41–11.45) and 10.52 Gy_*α*/*β*=3_ (95% CI: 9.16–11.88) in the first and second fraction ICBT, respectively. The absolute dose variation for bladder and rectum for various subvolumes between the two fractions is given in the Table 1.

**Figure 2:**
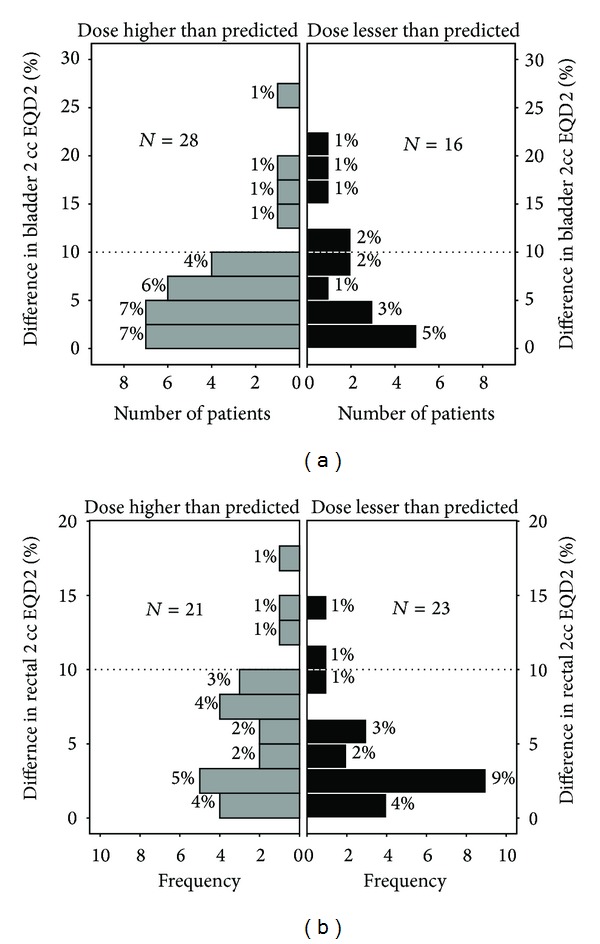
Population pyramids showing the difference in the predicted cumulative EQD2 (calculated assuming the doses were same in both fractions) from the actual cumulative EQD2. The ordinate axis represents the number of patients while the abscissa represents the percent difference between the hypothetical cumulative EQD2 and the actual cumulative EQD2. (a) and (b) show the population pyramids for bladder and rectum respectively. Grey bars represent the number of patients who had an increase in the actual cumulative EQD2 *D*_2 cc_ over that predicted if the dose in each fraction was considered to be same. The frequency of patients in whom the actual EQD2 would have been 10% more than the predicted is represented by the gray bars above the dotted line.

As can be seen from Figure 2(a) 28 patients (63.6%) received a higher dose to the bladder in the second fraction. Three patients had an actual cumulative EQD2 more than 90 Gy_*α*/*β*=3_, which would not have been recognized if the doses of the first fraction were considered to have remained the same in the subsequent fraction. Thus, 6.8% of the patients would have had a bladder dose exceeding the tolerance in absence of the second fraction CT.

As can be seen from Figure 2(b) 21 patients (47.7%) received a higher dose to the rectum in the second fraction. Two patients had an actual cumulative EQD2 more than 75 Gy_*α*/*β*=3_, which would not have been recognized if the doses of the first fraction were considered to have remained the same in the subsequent fraction. Thus, 4.5% of the patients would have had a bladder dose exceeding the tolerance in absence of the second fraction CT.

**Figure 3:**
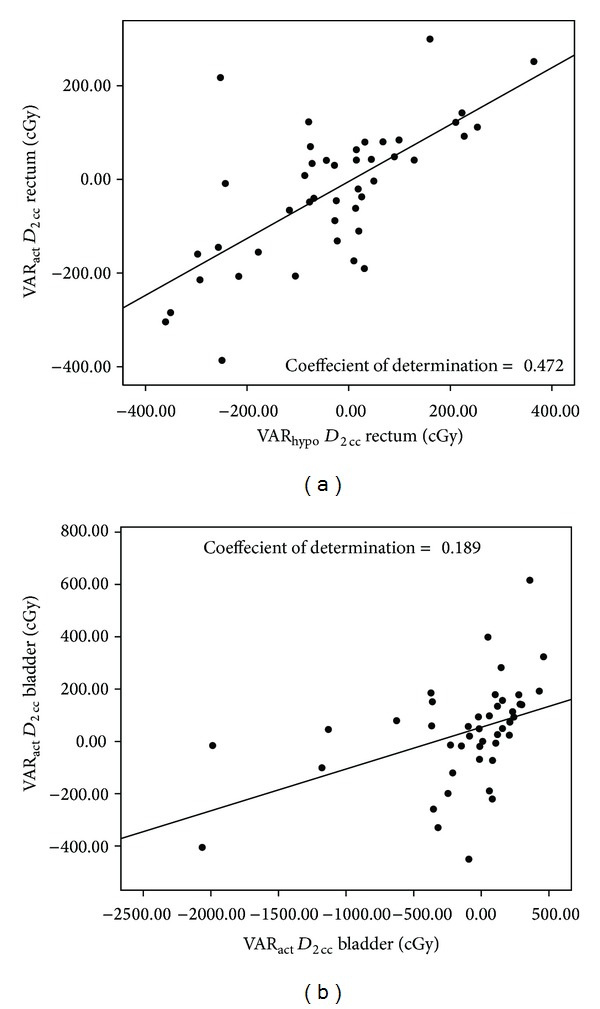
Showing the scatter plots of the actual variation in the organ doses (VAR_act_) versus the predicted variation (VAR_hypo_) due to changes in applicator placement/geometry alone. (a) is for Rectum and (b) is for Bladder. The superimposed linear regression line and the coefficient of determination are also shown. All doses are in cGy.

Regression plots in Figure 3 show how well the VAR_hypo_ (due to applicator position and geometry variations alone) could predict the VAR_act_ for rectum (a) and bladder (b). As seen VAR_hypo_ alone could predict about 47% of the VAR_act_ in the rectum and 19% of the VAR_act_ in the bladder. The remaining variation was due to the organ deformation related dose variations between the two fractions.

## 4. Discussion

HDR ICBT has several advantages in terms of allowing once daily treatment on outpatient basis, shorter treatment time, optimization of dose, better applicator design, and less tissue trauma during insertion. Clinical results have so far shown that HDR ICBT is equivalent to LDR ICBT in cervical carcinoma in terms of local control and late toxicities [12]. However, the disadvantage is the need for a fractionated delivery which results in interfraction variations in dose. The spatial variations in the position and geometry of the applicator in the pelvis, tumor regression during treatment, and organ deformation or movement are all factors which result in these variations.

Differences to the tune of 30 cm^3^ were seen in the bladder volume between the two fractions, but these differences were not statistically significant. The magnitude of variation in the rectal volume was quantitatively lesser and statistically insignificant between the two fractions.

Table 2 summarizes the results of series utilizing a volumetric imaging technique to quantify the magnitude of variation in dose to the bladder and rectum [10, 13, 14]. The use of the first fraction plan may result in dose variations as high as 60% and 29% of the prescribed dose in the bladder and rectal *D*_2 cc_, respectively [13, 14]. In their study, using MRI based planning, Kirisits et al. kept a cumulative dose constraint of EQD2 of 75 Gy_*α*/*β*=3_ and 90 Gy_*α*/*β*=3_ for the rectum and bladder, respectively [14]. They found in their series that three patients (21%) would have exceeded the constraints to either of these organs if the event subsequent fractions were not imaged. We observed that the same constraints would have been exceeded in seven patients (16%) if imaging was not performed in the second fraction.

In the past several authors have utilized orthogonal radiograph based planning to describe variations in applicator geometry and placement in HDR ICBT [2–9, 15]. Most of these authors have quantified the variations in terms of various angles and distances of fixed points on the bony pelvis from the applicator [2, 3, 5, 8, 9]. However this method does not give a true description of the actual moments and rotations that have occurred, as they are dependent on the frame of reference selected. For this reason also results from one series are difficult to compare against each other. The ideal method to quantify applicator geometry variations would be to apply a deformable image registration and describe the movement in terms of the transform applied for the fusion. However deformable image registration is still a developing technique and we lacked a software capable of deformable registration.

Clinicians would be more interested in the consequences arising from the applicator variation, namely, the dose variation in the OAR. In addition it would be of interest to evaluate how much of this dose variation is actually due to the applicator displacements. Rigid image registration allowed us to get a VAR_hypo_ that would occur if only the applicator varied between the applications. As can be seen, using this method about 47% of the VAR_act_ in the rectal dose and only 19% of the actual bladder dose variation can be explained. This implies that rest of the variation is due to the organ volume and shape changes.

The authors realize that in addition to these two factors optimization of the dose distribution would result in interfraction dose variations in the OAR. In the present study as the same loading pattern was used for both fractions without any optimization, the impact of the optimization was not observed. It is noteworthy that variable optimization in successive fractions is done mainly to account for the changes in the applicator placement/geometry or organ/target deformation and thus actually is dependent on these two.

Rigid image registration taking the bony anatomy as the frame of reference may seem counterintuitive in the present day era of three-dimensional image based brachytherapy. In the present study however this form of image registration allowed us to generate a hypothetical situation where only the applicator geometry/placement was varying between the two fractions. Use of rigid registration using the applicator as the frame of reference would also have given us a composite variation arising out of organ deformation and applicator placement/geometry variations. Also organ deformations for bladder and rectum depend on the placement/geometry variations to some extent (e.g., differing volume of vaginal packing resulting in organs being displaced to a different extent in subsequent fractions). Therefore the use of the registration technique described here is mandatory for isolating the influence of each of these two factors. Use of a deformable image registration technique would have negated the impact of organ deformation on the other hand.

The implication is that interfraction variation in the organ doses cannot be controlled by standardization of the application technique only. Changes in organ volumes and shapes are potentially more important determinants of their own dose variation. Controlling bladder volume may be possible by using a fixed bladder filling during the procedure; however, the shape changes that occur in the bladder are extensive. Davidson et al. have reported that despite the use of standardized bladder filling significant variations in the bladder dose can occur (Table 2) [13]. Therefore imaging needs to be repeated with each fraction in order to properly quantify the dose to the OAR.

In the present study we have not evaluated the dose variations in the target. Without the use of CT compatible applicators, target volume definition on CT images is fraught with uncertainties due to extensive artifacts encountered. In addition to the purpose of this study optimization of the dose distribution in the first fraction was not performed. Optimization of the dose distribution is likely to lead to higher variations in the dose distribution if the first fraction loading pattern is used in the subsequent fraction as highlighted by Kirisits et al. [14].

This leaves us with the important question as to how the interfraction variations can be reduced in practice. Part of the problem can be solved by using GA during the procedure which allows the best relaxation and packing. In addition every effort should be made to ensure that the same applicator type can be used in each fraction. Use of rigid or fixed applicators may also result in reduced variations as the applicator geometry can be maintained between fractions [13]. In our series all patients were treated after completion of external beam radiation which minimized the influence of tumor regression on the dose variation. While organ shape changes are inevitable, the organ volume variations may be reduced by using a standardized bladder filling or emptying routine [13]. User experience and use of lesser number of fractions will also result in reduced dose variation, albeit at the cost of a higher dose per fraction for the latter.

Despite the use of volumetric imaging the evaluation of dose variability in between fractions has several lacunae [16]. It is quite likely that in presence of the extensive organ deformations different sub-volumes of the organ may be in the high dose region in successive fractions [17]. Simple summation of the dose volume histogram in successive fractions overlooks this important factor. In organs with larger deformations during fractions like bladder and sigmoid colon this may result in a lack of correlation between the dose volume parameters and the clinically observed toxicity [16]. Future studies should therefore attempt to evaluate this variation using deformable image registration.

## 5. Conclusions

In the present study there were no statistically significant variations in the volumes or doses of OAR between the two fractions. However a significant proportion of patients may have a higher dose to the OAR in the second fraction in the absence of individualized planning. This increase is likely to be more detrimental where higher doses per fraction are used. Variations in OAR doses may be caused by organ deformation and/or changes in applicator placement/geometry. This study highlights the fact that organ deformation plays a greater role in determining the variation in interfraction dose as compared to changes in applicator placement/geometry. Thus majority of dose variation in HDR brachytherapy cannot be controlled and individualized imaging of fractions is necessary to accurately calculate the individual OAR doses.

## Figures and Tables

**Table 1:** Dose variation in the rectal and bladder doses in mean and 95% confidence intervals of mean.

	Bladder		*P* value	Rectum		*P* value
	Mean	95% CI		Mean	95% CI	
*D*_max⁡_	5.04	3.02–7.06	0.65	2.18	1.62–2.74	0.39
*D*_0.1 cc_	3.21	2.20–4.21	0.82	1.79	1.35–2.22	0.41
*D*_1 cc_	1.83	1.33–2.34	0.29	1.32	1.01–1.64	0.33
*D*_2 cc_	1.46	1.05–1.88	0.27	1.16	0.88–1.44	0.28

All values are in Gy. 95% CI: 95% confidence intervals of mean. *P* value is measured for difference in mean doses between two sessions using paired *t*-test. *D*_max⁡_ is the maximum point dose for the organ. *D*_0.1 cc_, *D*_1 cc_, and *D*_2 cc_ represent the minimum dose to 0.1, 1, and 2 cc volumes, respectively, in the most irradiated tissue adjacent to the applicator.

**Table 2:** Table showing variation in dose to rectum and bladder in various studies.

Author	Imaging	*N*	Applicator type	Prescribed dose	Mean variation rectal *D*_2 cc_	Mean variation bladder *D*_2 cc_
Patel et al. [12]	CT	69	TR	4.2 Gy	1.08 Gy (25.76%)*	1.22 Gy (29.00%)*
Davidson et al. [13]	CT	108	TR/TO	7 Gy	1.70 Gy (24.30%)	4.40 Gy (61.00%)
Kirisits et al. [14]	MRI	62	TR	7 Gy	3.50 Gy_3_^†^ (29.10%)	4.20 Gy_3_^†^ (24.70%)
Present series	CT	88	TO	9 Gy	1.16 Gy (12.90%)	1.46 Gy (16.20%)

All % variations are with respect to the prescribed dose to point A for the study. Mean variations in minimum dose to 2 cc volume in the most irradiated tissue adjacent to the applicator (*D*_2 cc_) for rectum and bladder are shown. Prescribed dose is prescribed dose per fraction. *N*: number of insertions studied; CT: computed tomography; MRI: magnetic resonance imaging; TR: tandem ring applicator; TO: tandem ovoid applicator; *dose to 95% volumes of the respective organs; ^†^dose in EQD2 assuming *α*/*β* ratio of 3.

## List of Abbreviations

ICBT: Intracavitary brachytherapy
HDR ICBT: High dose rate intracavitary brachytherapy
OAR: organs at risk
VAR_act_: Actual interfraction dose variations
VAR_hypo_: Hypothetical dose variation
*D*_2 cc_: Minimum dose to most irradiated 2 cc volume
